# Special Issue “Gut Microbiota in Disease Mechanisms and Therapy 3.0”

**DOI:** 10.3390/ijms27114866

**Published:** 2026-05-28

**Authors:** Dulcenombre Gómez-Garre, Javier Modrego

**Affiliations:** 1 Cardiovascular Risk and Microbiota Laboratory, Hospital Clínico San Carlos-Instituto de Investigación Sanitaria San Carlos (IdISSC), 2ª Planta-Norte, C/Profesor Martín Largos, s/n, 28040 Madrid, Spain; 2 Physiology Department, Faculty of Medicine, Universidad Complutense de Madrid (UCM), Plaza Ramón y Cajal s/n, 28040 Madrid, Spain; 3 Biomedical Research Networking Center in Cardiovascular Diseases (CIBERCV), Instituto de Salud Carlos III, Av. Monforte de Lemos, 3-5, 28029 Madrid, Spain; 4 ImFINE Research Group, Department of Health and Human Performance, Universidad Politécnica de Madrid (UPM), C/Martín Fierro, 7, 28040 Madrid, Spain

## 1. Introduction

The human gut microbiota, a dynamic and highly complex ecosystem comprising bacteria, archaea, fungi, viruses, and their collective genetic material, has emerged over the past two decades as one of the most intensively studied determinants of human health and disease [1,2]. Harboring an estimated 100 trillion microorganisms, predominantly from the phyla Firmicutes and Bacteroidetes, the gut microbiota operates as a metabolically active “organ” with far-reaching effects on physiology that extend well beyond the gastrointestinal tract [3]. Through the production of bioactive metabolites, modulation of immune responses, and maintenance of intestinal barrier integrity, the microbiota participates in a continuous and bidirectional dialogue with its host that is essential for homeostasis [4].

Central to this dialogue are short-chain fatty acids (SCFAs), acetate, propionate, and butyrate, generated by anaerobic bacterial fermentation of dietary fiber. SCFAs serve as the primary energy source for colonocytes, reinforce epithelial tight junction integrity, and exert potent immunomodulatory effects through activation of G protein-coupled receptors and inhibition of histone deacetylases, thereby regulating cytokine production, T regulatory cell differentiation, and systemic inflammatory tone [5]. Reductions in SCFA-producing taxa such as *Faecalibacterium prausnitzii*, *Roseburia*, and *Bifidobacterium* have been consistently observed across a wide range of disease states, underlining the centrality of these metabolites to microbial health functions [6].

When the composition or functional capacity of the gut microbiota is disrupted, a state called dysbiosis, the consequences extend across multiple organ systems. The main pathogenic mechanisms involve impaired intestinal mucosal barrier function, chronic low-grade inflammation, immune dysregulation, and metabolic abnormalities, collectively operating through interconnected inter-organ communication networks including the gut–brain, gut–liver, gut–lung, and gut–cardiovascular axes [7]. Through these pathways, dysbiosis has been implicated in the pathogenesis of metabolic disorders such as obesity and type 2 diabetes, cardiovascular diseases including hypertension and atherosclerosis, neurodegenerative conditions such as Alzheimer’s and Parkinson’s disease, immune-mediated disorders, and cancer. Microbial metabolites such as trimethylamine N-oxide (TMAO) and secondary bile acids have been identified as key mediators linking gut microbial activity to cardiovascular and metabolic risk [4,8], while alterations in tryptophan metabolism and SCFA signalling along the gut–brain axis have been associated with neuroinflammation and neurodegeneration [9,10,11].

The growing recognition that the microbiota is not merely a bystander but an active participant in disease pathogenesis has stimulated parallel interest in therapeutic strategies targeting microbial communities. Dietary interventions, prebiotics, probiotics, postbiotics, and fecal microbiota transplantation (FMT) are being actively explored as tools to restore microbial balance and, thereby, mitigate disease. Nevertheless, important challenges remain [12,13,14,15]. Establishing causality rather than mere association is methodologically demanding; interindividual variability in microbiota composition complicates the generalization of findings; and the translation of experimental and observational insights into reproducible, evidence-based clinical interventions has proven difficult. The field also faces the challenge of moving beyond broad taxonomic profiling toward a mechanistic understanding of specific microbial functions and their context-dependent effects on host biology.

This Special Issue brings together seven contributions that address these challenges from complementary perspectives, spanning conditions that range from early infancy to critical illness and from gastrointestinal disorders to cardiovascular and post-infectious disease. Together, they reflect both the breadth and the increasing mechanistic specificity of contemporary gut microbiota research.

## 2. An Overview of Published Articles

The issue opens by focusing on a common but frequently misdiagnosed condition. Wielgosz-Grochowska et al. (contribution 1) examined whether the different small intestinal bacterial overgrowth (SIBO) subtypes, hydrogen-predominant, methane-predominant, and mixed, affect nutritional status and dietary intake differently. In a cohort of 67 newly diagnosed patients, they identified subtype-specific associations with vitamin D, ferritin, and fiber deficiencies, suggesting that SIBO subtyping should be integrated into nutritional assessment and treatment planning. This work reinforces the idea that microbiota-related pathology is not monolithic and that precise approaches are required even within the same diagnostic category.

Regarding the earliest stages of life, Szczuko et al. (contribution 2) investigated the relationship between SCFA concentrations and gastrointestinal symptoms in infants aged 1, 3, 6, and 12 months. Using gas chromatography to analyze fecal samples, they observed that elevated levels of butyric and valeric acids were associated with flatulence and bloating during the first few months of life, while changes in acetic and isovaleric acids correlated with defecation problems. These findings highlight the functional and symptomatic relevance of microbial metabolite profiles, even in early childhood.

The role of radiation exposure in reshaping microbial communities is addressed by Chakraborty et al. (contribution 3), using an integrative metagenomic and metabolomic analysis of the descending colon contents in mice subjected to total body irradiation. Their results revealed significant, sex-biased alterations in microbial composition and SCFA-related metabolic networks, with more pronounced and persistent dysbiosis observed in male mice than in females. These findings have direct implications for the design of radiation countermeasures and suggest that sex should be considered a key variable in microbiota-focused therapeutic strategies.

The therapeutic dimension of the problem is further developed in the prospective pilot study by Chernevskaya et al. (contribution 4), who evaluated a microbiota-focused treatment strategy in patients with chronic critical illness (CCI), stratified according to the severity of microbial dysfunction. By applying sequential regimens that included metabiotics, enteral nutrition, and antibiotics safe for anaerobic bacteria, they demonstrated improvements in neurological status, inflammatory markers, and a significant reduction in the incidence of nosocomial pneumonia. This study provides preliminary but compelling evidence that systematically addressing gut microbiota dysfunction in the CCI can improve clinical outcomes beyond what conventional organ support strategies can achieve.

Junka et al. (contribution 5) addressed a more specific question: whether octenidine-containing lozenges, used as a topical treatment for pharyngeal infections, pose a risk to the gut microbiota. After evaluating antimicrobial and antibiofilm activity against 106 microbial strains under physiologically relevant conditions, they demonstrated that octenidine effectively eliminated oropharyngeal pathogens without affecting the commensal gut microbiota. This work contributes to the growing body of evidence underscoring the need to assess the microbiota safety profile of antimicrobial agents as a fundamental part of their clinical development.

The intersection of microbiota and immune-mediated post-infectious disease is addressed by Caliman-Sturdza et al. (contribution 6), who synthesized current evidence on gut and oral microbiota alterations in long COVID. Patients with post-acute sequelae of SARS-CoV-2 infection consistently show reduced microbial diversity, a decrease in SCFA-producing taxa such as *Faecalibacterium prausnitzii* and *Bifidobacterium*, and an enrichment of pro-inflammatory species. The authors propose that these microbial profiles may maintain systemic inflammation and neurological symptoms through dysregulation of the gut–brain and gut–lung axes, and they review preliminary evidence supporting microbiome-targeted therapies, including probiotics and fecal microbiota transplantation, as complementary strategies in the management of long COVID.

Finally, the metabolic and systemic reach of gut microbiota dysfunction is illustrated by Neag et al. (contribution 7), who reviewed the relationship between gut microbiota composition and the development and progression of cardiovascular disease. Their work highlights how microbial metabolites, including SCFAs, TMAO, and bile acids, modulate vascular inflammation, endothelial function, and lipid metabolism. Importantly, the authors analyze the potential of nutraceuticals to positively remodel the microbiota and, therefore, reduce cardiovascular risk, positioning dietary intervention as a viable and underexplored therapeutic approach.

## 3. Conclusions

Taken together, these contributions reflect the remarkable scope of gut microbiota research and its clinical relevance in diverse pathological contexts. Looking ahead, future work should prioritize longitudinal studies capable of establishing causality, the development and validation of microbiota-based biomarkers for clinical stratification, and the design of precision therapeutic strategies that take into account interindividual microbial variability, age, sex, and disease context. We sincerely thank all the authors and reviewers for their valuable contributions to this issue.

## Data Availability

The raw data supporting the conclusions of this article will be made available by the authors on request.
